# The use of medicinal plants in the trans-himalayan arid zone of Mustang district, Nepal

**DOI:** 10.1186/1746-4269-6-14

**Published:** 2010-04-06

**Authors:** Shandesh Bhattarai, Ram P Chaudhary, Cassandra L Quave, Robin SL Taylor

**Affiliations:** 1 Nepal Academy of Science and Technology, Khumaltar, Lalitpur, Nepal; 2 Central Department of Botany, Tribhuvan University, Kirtipur, Kathmandu, Nepal; 3 University of Arkansas for Medical Sciences, College of Medicine, Little Rock, AR, USA; 4 Community Medicine, Queens University, Kingston, Ontario, Canada

## Abstract

**Background:**

This study documents the use of medicinal plants from the Mustang district of the north-central part of Nepal. Traditional botanical medicine is the primary mode of healthcare for most of the population of this district and traditional Tibetan doctors (*Amchi*) serve as the local medical experts.

**Methods:**

Field research was conducted in 27 communities of the Mustang district in Nepal from 2005-2007. We sampled 202 interviewees, using random and snowball sampling techniques. After obtaining prior informed consent, we collected data through semi-structured interviews and participant-observation techniques. Voucher specimens of all cited botanic species were deposited at TUCH in Nepal.

**Results:**

We recorded the traditional uses of 121 medicinal plant species, belonging to 49 vascular plant and 2 fungal families encompassing 92 genera. These 121 species are employed to treat a total of 116 ailments. We present data on 58 plant species previously unknown for their medicinal uses in the Mustang district. Of the medicinal plants reported, the most common growth form was herbs (73%) followed by shrubs, trees, and climbers. We document that several parts of individual plant species are used as medicine. Plant parts were generally prepared using hot or cold water as the 'solvent', but occasionally remedies were prepared with milk, honey, jaggery, ghee and oil. *Amchis *recommended different types of medicine including paste, powder, decoction, tablet, pills, infusion, and others through oral, topical, nasal and others routes of administration.

**Conclusions:**

The traditional pharmacopoeia of the Mustang district incorporates a myriad of diverse botanical flora. Traditional knowledge of the remedies is passed down through oral traditions and dedicated apprenticeships under the tutelage of senior *Amchi*. Although medicinal plants still play a pivotal role in the primary healthcare of the local people of Mustang, efforts to ensure the conservation and sustainable use of medicinal species are necessary.

## Background

Plants and plant products are the primary source of medicine and a highly valued resource in Nepal. Plant constituents continue to be a vital part of Western medicine, and are still considered an important source of novel compounds in the field of drug discovery [1]. There are between 35,000 and 70,000 plant species that have been used for medicinal purposes in the world [2], and about 6,500 species of which occur in Asia [3]. In Nepal, at least 1,600 to 1,900 species of plants are commonly used in traditional medicinal practices [4,5].

Traditional medicine in Nepal is used extensively by majority of the population, and includes Ayurveda, traditional Chinese medicine (TCM), Unani and various forms of indigenous medicine including Tibetan *Amchi *medicine [6-9]. Traditional medicine in Nepal comprises those practices based on beliefs that were in existence often for hundreds to thousands of years before the development and spread of modern medicine, and which are still in use today [10]. In the past in many rural areas of Nepal, traditional medicinal knowledge and practice was passed down entirely via oral tradition based on a lineage mode of transmission and personal experience [11]. More recently, however, knowledge transfer has also occurred through formally recognized school level education [12-14].

Approximately 90% of the Nepalese people reside in rural areas where access to government health care facilities is lacking [11,15]. It is estimated that there is a ratio of one physician for 6,500 (1:6500) people and one healer for fewer than 100. The physician to population ratio of Nepal is lower then that of India (1:2000), Bangladesh (1:3500 respectively), and Sri Lanka (1:4500) [16,17]. In Nepal, a total of 4,088 government health posts have been established and the ratio of health posts to the population it serves (1:5663) is very low. The Mustang district, where field research was conducted, has 17 health posts for a total of 14,981 people. While the health post to population ratio (1:881) in the Mustang district is better much than the national average (1:5663) [18], the remote location and rugged terrain do not permit easy access to these facilities. Due to these issues of accessibility and other socioeconomic and cultural factors, local people rely more heavily on traditional forms of medicine.

The Mustang district covers 3,639 sq. km [19] and is located in the trans-Himalayan Arid Zone, in the Mid-Western Development region of north-central Nepal and is bounded to the south by Myagdi, to the west by Dolpa, to the east by Manang, and to the north by the Tibetan Autonomous region of the Peoples Republic of China. The district lies within the Annapurna Conservation Area Project (ACAP), which covers five districts and is the largest protected area in Nepal covering 7629 sq. km [19]. It is ranked in 17^th ^position on the socioeconomic and infrastructural development index, 22^nd ^in the Health and Development Index and 42^nd ^in the Health institutions density among 75 districts in Nepal [20]. The Mustang district is mountainous with fragile ecosystems where local biodiversity plays an important role in meeting the basic daily needs of the indigenous peoples inhabiting this region.

The vegetation of Mustang has been categorized into 8 types namely: mixed forest (*Pinus wallichiana *forest, *Betula utilis *forest, *Hippophae salicifolia *forest, *Caragana gerardiana *forest, *Caragana gerardiana *and *Lonicera spinosa *forest, *Juniperus *forest) and grasslands with pure stocks of Poaceae [21]. The area is characterized by the high altitude, cold climate, semi-desert environment [22], with altitudinal variations of 1,500 to 8,000 m.a.s.l. The district has characteristic vegetation with a freezing season of about 73-119 days (Marpha-Lo-Manthang) [23], and is dominated by shrubby and dwarf plant communities [24]. The influence of such characteristic environmental conditions in the Himalayan region including Mustang established favourable growth conditions for some of the medicinal plant species at altitudes as high as 6000-6300 m.a.s.l. [14].

Documentation of ethnobotanical knowledge of unique plant species has gained importance in the remote arid trans-Himalayan region of Nepal (Mustang, Manang, Dolpa, and Tibet), which have similar geography and bioclimate [25-41]. It has been estimated that between 246-310 species of flowering plants are endemics to Nepal and the great majority of these species are located in Mustang (78 species) [10,42]. Many of these plants have been used by local indigenous people for centuries, with medicinal uses playing an important role in both health and culture.

Research has shown that the Mustang district is an important area for many useful plant resources [13,43-46] and the district has not been adequately explored. We chose the Mustang district for this ethnomedicinal study for the following reasons: (i) The study area is rich in diversity of medicinally used plant species [13,47]; (ii) The society (communities) possess rich traditional knowledge (i.e. cultural diversity) [13,48]; and (iii) There is a culture of tradition in which healers or knowledgeable persons transmit their traditional knowledge from generation to generation, usually through apprentices [13]. In this study, we aimed to address the following questions: (a) How are plant resources being used by the local communities in primary health care? (b) Are the indigenous people involved in conservation activities? (c) How is the traditional knowledge of indigenous people transmitted, conserved and utilized?

In remote villages of Mustang, traditional medicines are of great importance in the primary healthcare of indigenous people due to the lack of sufficient and reliable government health facilities and modern Western medicines. Therefore, local plant resources are the principal source of medicine, and are prescribed by traditional healers as medicines. However, loss of biodiversity in Nepal due to several factors may also contribute to the loss of valuable indigenous knowledge of plants of several indigenous communities in Nepal [49,50], including Mustang. To overcome this problem, we have undertaken this ethnobotanical research project with the aim of documenting medicinal plant uses and the associated indigenous knowledge of local people of Mustang.

## Materials and methods

### Plant collection and identification

**Figure 1:**
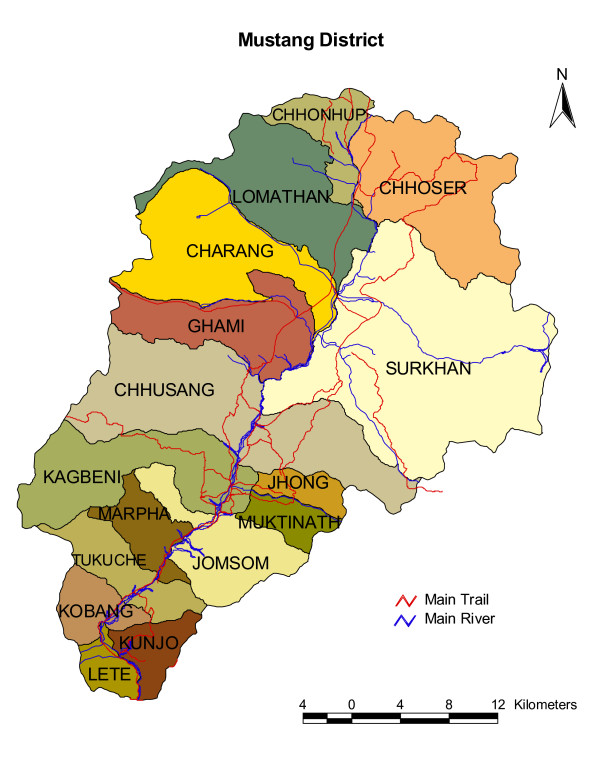
**Mustang District, Nepal, site of three ethnobotanical field visits from 2005-2007**.

The plants were collected in and around the villages of Ghasa (2010 m), Lete (2480 m), Sekung Taal (2620 m), Larjung (2550 m), Kalopani (2510 m), Tukuche (2950 m), Kobang (2640 m), Kokhethanti (2520 m), Marpha (2670 m), Jomsom (2720 m), Thini (2800 m), Kagbeni (2810 m), Eklebhatti (2740 m), Jharkot (3270 m), Mukthinath (3300 m), Chhuksang (2940 m), Chele (3050 m), Samar (3660 m), Syangboche (3820 m), Ghemi (3490 m), Dhakmar (3535 m), Ghiling (3510 m), Tamagaon (3480 m), Jhaite (3570 m), Bhena (3690 m), Tsarang (3620 m) and Lomanthang (3720 m), in the Mustang district of Nepal from 2005-2007 (Figure 1).

Plants were selected based on of their use by inhabitants of Mustang. Only species that were consistently used to treat the same illness by several healers and villagers were selected. Voucher specimens were made for each species collected in this study and deposited in the Tribhuvan University Central Herbarium (TUCH), Kathmandu, Nepal. Plants were identified by two of us (SB and RPC) with the help of standard botanical literature [51-55]. Nomenclature of the identified species follows standard literatures [56-61] and plant family assignments follow the current Angiosperm Phylogeny Group [62]. Voucher specimens were also cross-checked with previously collected herbarium specimens from the Manang district that had been previously identified by the National Herbarium and Plant Laboratories, Godawari, Lalitpur (KATH) and the Royal Botanic Garden (RBGE), Edinburgh, United Kingdom.

### Study Participants and Interviews

**Figure 2:**
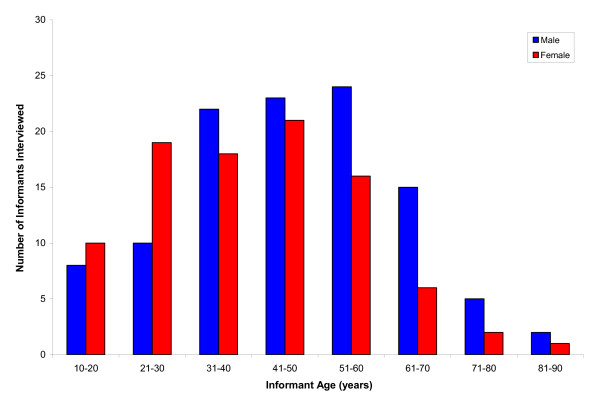
**Study participants interviewed (villagers, healersand *Amchis*)**.

Consent for this research project was obtained in writing from the Annapurna Conservation Area Project (ACAP), Pokhara, and prior informed consent (PIC) was obtained verbally from each participant before they were interviewed. The project was approved by the Central Department of Botany Research Committee of Tribhuvan University. We followed the ethical guidelines adopted by the ICE/International Society of Ethnobiology. We met with local community elders to explain the research project methods and intent and initiated participant recruitment only after approval by these community leaders. We employed random and snowball sampling techniques to identify potential participants and interviewed a total of 202 people (109 men and 93 women) representing a sample of the population across age groups (Figure 2).

Study participants included individuals from various ethnic and socioeconomic backgrounds, including *Amchi *healers, medicinal plant traders, farmers, hotel or shop owners or managers, footpath traders, homemakers, and village elders. However, the traditional senior and junior Tibetan doctors (*Amchis*) were the key source of information regarding medicinal plant use. The traditional healers of Mustang comprise senior *Amchis *(Tibetan medicinal practitioners who see patients in their clinics and teach students in medical school), junior *Amchis *(student studying *Amchi *medicine), plant traders (traders of Mustang medicinal plants in major cities of Nepal), and knowledgeable villagers (including herders, farmers, hotel owners etc). The local knowledgeable villagers, healers and *Amchis *interviewed who consented to have their names and knowledge published are listed in the acknowledgements. The completed manuscript created as a result of this project will be returned to the participating communities, and copies given to key people who collaborated in the research.

Interviews were conducted in the local Nepali or in Gurung language dialect (translated by an interpreter), and data was collected by direct and participant-observation of study participants in fields and forests and through semi-structured interviews. Interview protocols and field observations all followed standard ethnobotanical methods [63-65]. *Amchis *who were influenced by deep practical knowledge of medicinal plants of Mustang district were interviewed during July 2005, September 2006 and June 2007. A total of 75 days were spent in the study sites. At first interviews were conducted using the 'specimen display' method. After collecting plant specimens for research, we showed these fresh specimens to the locals in order to elicit information. The same plant specimens were shown to different people to confirm the accuracy of the results. When convenient to the participants, they were asked to accompany the researchers for a walk, allowing for both plant collection and detailed information gathering. A consensus index was created based on agreement in the medicinal use of the different species cited and is included in Additional File 1.

## Results and Discussion

A total of 121 species belonging to 49 vascular plant and 2 fungal families and 92 genera were reported and are indicative of the rich diversity of medicinal plant species found in this area. Study results are presented in alphabetical order by family and then followed by scientific name, voucher number, local vernacular names (Gurung/Thakali/amchi/Nepali), phenology, and detailed uses - including methods of preparation, dosage and administration of medicine (Additional File 1). The largest number of medicinal species came from Asteraceae (18), which was followed by Ranunculaceae (8), Rosaceae (7), Lamiaceae (6), and Fabaceae (5) (Table 1).

**Table 1:** Division of medicinal plants and fungi documented by family designation.

Families	Species	Proportion	Families	Species	Proportion
Alliaceae	3	2.48%	Malvaceae	1	0.83%
Amaranthaceae	1	0.83%	Menispermaceae	1	0.83%
Apiaceae	2	1.65%	^§^Morchellaceae	1	0.83%
Araceae	2	1.65%	Nyctaginaceae	1	0.83%
Asparagaceae	1	0.83%	Orchidaceae	1	0.83%
Asteraceae	18	14.88%	Orobanchaeae	1	0.83%
Berberidaceae	4	3.31%	Papaveraceae	3	2.48%
Betulaceae	1	0.83%	Phrymaceae	1	0.83%
Bignoniaceae	1	0.83%	Pinaceae	1	0.83%
Boraginaceae	3	2.48%	Plantaginaceae	2	1.65%
Brassicaceae	3	2.48%	Polygonaceae	4	3.31%
Cannabaceae	1	0.83%	Primulaceae	3	2.48%
Caprifoliaceae	2	1.65%	Ranunculaceae	8	6.61%
Chenopodiaceae	1	0.83%	Rosaceae	7	5.79%
^§^Clavicipitaceae	1	0.83%	Salicaceae	1	0.83%
Convolvulaceae	1	0.83%	Saxifragaceae	2	1.65%
Crassulaceae	1	0.83%	Scrophulariaceae	1	0.83%
Cupressaceae	4	3.31%	Solanaceae	4	3.31%
Elaegnaceae	2	1.65%	Tamariaceae	1	0.83%
Ephedraceae	1	0.83%	Taxaceae	1	0.83%
Ericaceae	2	1.65%	Thymelaeaceae	1	0.83%
Fabaceae	5	4.13%	Urticaceae	1	0.83%
Gentianaceae	2	1.65%	Valerianaceae	2	1.65%
Juglandaceae	1	0.83%	Violaceae	1	0.83%
Lamiaceae	6	4.96%	Zingiberaceae	1	0.83%
Liliaceae	1	0.83%	**TOTAL**	**121**	**100%**

^§ ^Indicates fungal family.

These 121 species were found to treat 116 ailments (Table 2), 92 of which were used to treat more than one disease and the remaining 29 species were used to treat only one disease (Additional File 1). We have added 58 new medicinal plants, noted with asterisks (Additional File 1), in addition to the previous works of [13,44,47]. Many of these newly added plant species for Mustang are popular throughout Nepal and are used to treat a broad spectrum of ailments [12,15].

**Table 2:** Aliments included in each illness category.

Illness category	Ailments
Dermatological	Allergy, boils, allergic skin, skin diseases, warts

Fever	Fever, chronic fever, lung fever, malarial fever

Gastrointestinal	Bile diseases, bile disorders, liver diseases, stomach diseases, diarrhoea, dysentery, gastritis, constipation, intestinal worms, anthelmintic, stomach swelling

General health	Chronic diseases, inappropriate medication, to counteract the effect of poison, anti-poison, food poisoning, hair long, hair black, gingivitis, mouth swelling, snake bite, vomiting, to remove lodged bones or spines, dehydration, cancer, eye diseases, poor vision, bone fractures, joint swelling, typhoid, diseases of air, diseases of wind, vitamin, tonic, nutritious, loss of appetite, periods of fatigue, low energy, edema, nose swelling, burns, cold, cuts, child birth, over flow of blood in menstruation, over flow of blood in child birth, menstrual disorders, pregnant women, nerve diseases, nerve dispersed condition, communicable diseases, bone spurs, hemorrhoids, pneumonia, digestive, blood diseases, to increase blood, blood circulation, blood deficiency, blood purifier, reduces fats, thins blood in coagulation period, blood pressure, vertigo/dizziness, nose bleeding, pulse rate, tuberculosis, heart diseases, pain near the side of heart

Infections	Infected wounds, skin wounds, wounds, infection, infection diseases

Pain	Headache, stomachache, rheumatism, body pain, leg pain, backbone pain, joint pain, hand pain, ear pain, chest pain, ribs pain, bodyache, numbness of limbs, black worms of teeth, tooth pain, tootache

Respiratory	Cough, sinusitis, tonsillitis, chronic lung diseases, chronic respiratory diseases, respiration, asthma, bronchitis

Urinary	Kidney stone, Kidney diseases, red color urine, diseases of urine, painful urination, excessive urination, diuretic, difficulty in passing urine

Jaundice	Jaundice

**Figure 3:**
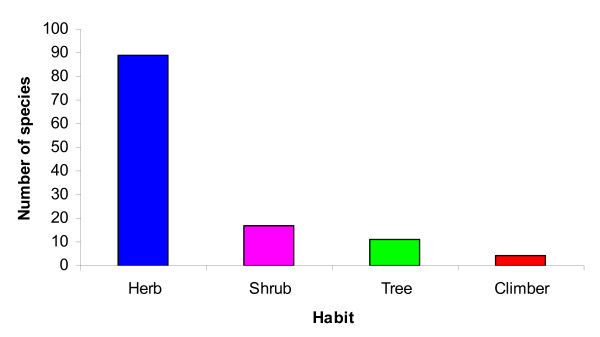
**Growth forms (habits) of the reported medicinal plant species**.

The study of the growth form of the medicinal plants revealed that herbs made up the highest proportion of medicinal plants represented 89 species followed by shrubs (17), trees (11), and climbers (4) (Figure 3). Similar results were also obtained from previous studies on the distribution pattern of life forms of medicinal plant species in the Nepalese Himalaya. Ghimire et al. 2008 [14] revealed that 45-70% of the total naturally growing species are long-lived herbaceous perennials followed by shrubs (16.6%), annual/biennial herbs (15.6%), tree (13.6%), woody climbers (6.5%), and herbaceous climbers (2.3%).

**Figure 4:**
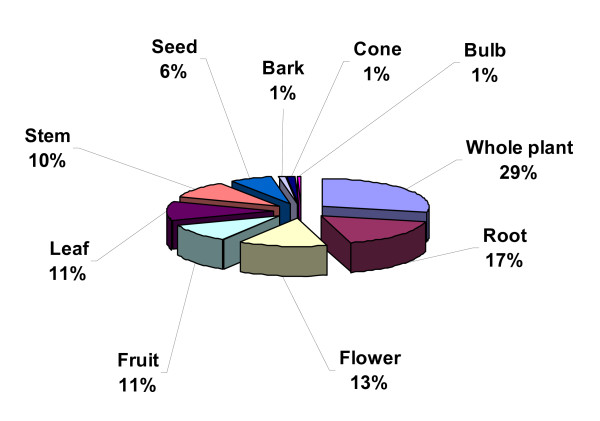
**Plant parts used in the preparation of medicine**.

This study recorded that several parts of individual plant species are used as medicine. The most commonly used medicinal plant part was the root (30 species), followed by flowers (23), fruits and leaves (19 each), stem (17), seed (11), bark and cone (2 each), and bulb (1). In addition to the above common parts used, whole plant (49 species, 29%), were commonly uprooted (Figure 4).

The most commonly selected plant parts may be preferential because such parts contain more active principles in comparison to the least used parts. Leaves, roots, stems and flowers are physically more vulnerable to attack by herbivores or pathogens than the hardier bark or cones and may contain more chemical defense compounds in the form of biologically active secondary metabolites. However, several studies have indicated that large scale harvesting of roots, leaves, stems and flowers can have a negative influence on the survival and continuity of useful medicinal plants and hence impacts sustainable utilization of plants [66].

**Figure 5:**
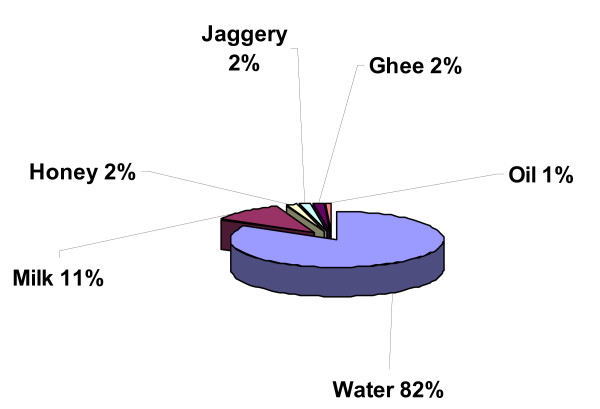
**Types of drug excipients used in the preparation of medicine**.

Plant parts were generally prepared using hot or cold water (100 species) as the solvent, but occasionally remedies were prepared with milk (14), honey (2), jaggery (gur - unrefined, whole sugar- 2 species), ghee (2) and oil (1) (Figure 5). The *Amchi *explained that water is a common, readily available, and cheap solvent and the good solubility of active components in water made it commonly used in the traditional medicine preparation. Other infusion materials such as milk, honey, oils, jaggery, and ghee are expensive and not always available when needed. In addition, milk, oil, honey and ghee may be used for their properties to dissolve active phytochemicals that are not water soluble.

**Figure 6:**
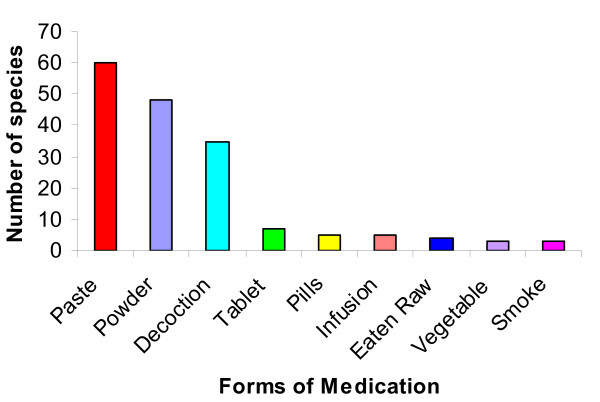
**Forms of medication used**.

*Amchis *recommended different forms of medication including paste (60 species), powder (48), decoction (35), tablet (7), pills (5), cold infusion (5), and others (Figure 6). This study often recorded the use of paste, powders and decoctions in comparison to tablet, pills and infusions. Most often standard medicines are prescribed in mixed ingredient form by mixing several valuable medicinal plants and additives. *Amchi *believe that using plant mixtures in the preparation of a medicine is important as a single plant alone may not be sufficient to cure any disease completely, whereas the combination of several medicinal plants increases the quality and efficacy of medicine. Similar observations have also been recorded amongst the Kani communities in India [67].

Certain zootherapeutic remedies, or medicines based on animal products, were also recorded. Some common animal products used in the creation of medicines included tortoise bones and the horn and urine of the Himalayan musk deer (*Moschus chrysogaster*). The *Amchi *traditional method of maintaining the good quality of herbal medicine is unique. For centuries, *Amchi *have been storing herbal medicine in a bag created from the skin of *M. chrysogaster *skin, which is tied twice with thread. Tying the herbal medicine in *M*.*chrysogaster *skin allows it to remain effective for one to two years. *Amchi *use a stone slab in place of the electric grinder in the preparation of medicine because they feel that heat created by the grinder may degrade the active chemical constituents of plant powder and thus reduce the quality of the medicine. The powder is then mixed with water and a sufficient amount of additives - honey, jaggery, etc. Additives are added according to the need of the specific plant powder to aid with the shape of the prepared pills (rounded, rectangular, etc). The process of boiling continues and complete evaporation of the water makes it easy to form the medicinal mixture into the preferred shape.

**Figure 7:**
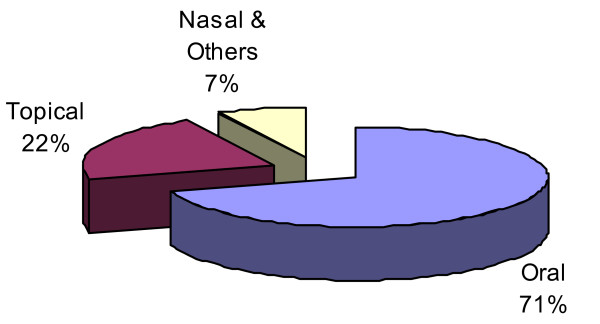
**Routes of administration of medicinal plants remedies**.

The medicinal plant preparations were administered to the local people of Mustang through different routes including oral, topical, nasal and others. Oral (115 species) was the most commonly used route of administration, and was followed by topical (36), nasal and others (12) (Figure 7). Similar observations have also been obtained in other ethnobotanical studies [66,68].

*Amchis *always collect local medicinal plants themselves. They stress that this is very important because they have extensive experience in the identification of Himalayan medicinal plants. They worry that a misnamed or falsely collected sample may be dangerous and cause the death of a patient. This is particularly true in the case of *Aconitum orochryseum *and *A*.*spicatum *(Ranunculaceae). *A*. *spicatum *is highly poisonous and is difficult to differentiate from *Aconitum orochryseum*. Using the wrong species by mistake can result in death. The medicine from this plant can only be prepared by highly experienced *Amchi*. Medicine must be made by *Amchi *with other medicinal plants of the Himalaya, so that the poison of that plant is inactivated without inactivating the other medicinal properties. In Mustang, *A*.*spicatum *whole plant (mainly root) is used to make medicinal tablets by the *Amchi *to treat infected wounds; as a tonic to provide relief from general weakness; to counteract the effects of poison, including inappropriate self medication, poison ingested on purpose or accidentally, poisonous animal stings or bites; boils; fever; allergy and edema. It is never used alone and is always mixed with several other medicinal plants of the Himalaya. A paste of the roots is applied for allergy, boils, cuts, wounds and edema after mixing with other medicinal plants.

**Figure 8:**
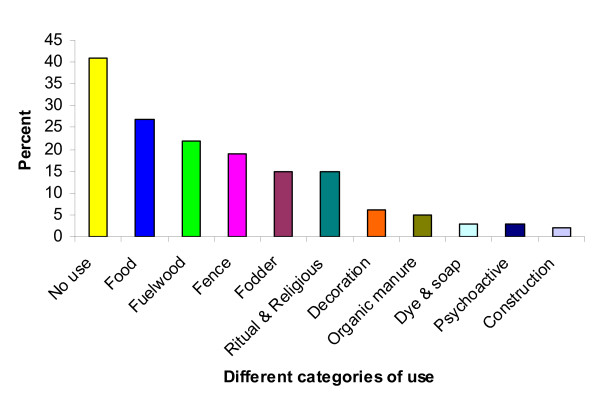
**Other uses of medicinal plant species**.

The 121 medicinal plant species recorded from Mustang were also used in miscellaneous categories of uses and hence such additional uses have added the value to medicinal plant species. The majority of medicinal plant species were used for food (33 species), fuelwood (27), fence (24), fodder and ritual & religious (19 each), decoration (8), organic manure (7), dyes & soap and psychoactive (3 each), and construction (2). However, many (50 species) have only medicinal use (Figure 8).

Most of the documented medicinal plant species were collected from wild habitats where very few species like *Allium carolinianum*, *Allium wallichii*, *Prunus armeniaca*, etc. were also cultivated for regular use. Medicinal plant species were used both in dried and fresh forms and are also collected and stored for future purpose. Autumn has been considered the best season for the collection of roots and spring for collection of stems. Important days or months for the collection of individual plants vary greatly and are known best by *Amchi*. The time of collection of plant parts for medicine is also very important in capturing the active principles. Therefore, *Amchi *strictly collect specific plant parts during a specific time and use them to prepare traditional medicine. Overall, the majority of locals consider *Amchi *medicines and medical system to be effective and local people have a deep faith in them.

Several medicinal plants including *Allium wallichii*, *Aconitum orochryseum*, *Cordyceps sinensis*, *Dactylorhiza hatagirea *and *Neopicrorhiza scrophulariiflora *are collected from the local habitat as a source of cash income. Such illegal mass collection of the important species is a type of unsustainable harvesting which leads to the exploitation from the natural habitat in the future. Therefore over-harvesting of important medicinal plants should be prohibited and monitored and immediate conservation and management approaches should be followed for the sustainable use of natural ecosystem of Mustang.

## Conclusion

Traditional *Amchi *medical practitioners maintain a great depth of knowledge on the subject of medicinal plants. Medicinal plants still play a pivotal role in the primary healthcare of the local people in the study area. Due to the lack of Western medicine, modern government health posts, difficult geography of the district as well as a strong cultural belief in the power of folk medicines, the *Amchi *system serves as a popular provider of primary healthcare in Mustang. Although such healthcare practices have been in place for centuries in Mustang, they are at risk of being lost to future generations. This is due primarily to changes in socioeconomics of the region as younger generations are eager to migrate outside the country for employment. The continued practice of training as an *Amchi *apprentice is necessary for the survival of this traditional medical knowledge.

Although local efforts to conserve medicinal plant resources are still inadequate, the long held traditional beliefs of the population regarding folk medicine has its own unintentional role in conservation, management and sustainable utilization. While over-harvesting of some important medicinal plants has increased, many *Amchi *are working towards both biological conservation of the medicinal plants through sustainable harvesting and protection of wild species and conservation of their cultural heritage. In some villages (Lete, Lomanthang, etc), people have started to conserve medicinal plants by domesticating them in home gardens, but these efforts make up only a small portion of measures necessary to conserve these species. The involvement of local communities and local stakeholders with effective monitoring of the local community resources will be the fundamental ingredient of *in situ *conservation of medicinal plants in Mustang. Over-harvested populations of medicinal plants from natural habitats can be improved with the aid of collaborative research projects between the local indigenous people and national and international partners having associated experts.

## Competing interests

The authors declare that they have no competing interests.

## Authors' contributions

Author SB performed the interviews with the healers, identified the herbarium specimens with RPC and drafted and finalized the manuscript with RPC, CLQ, and RSLT. Author RPC identified herbarium specimens with SB, supervised the research works and finalized the manuscript with SB. Author CLQ drafted and finalized the manuscript with SB. Author RSLT supervised the research works and drafted and finalized the manuscript with SB. All authors read and approved the final manuscript.

## Supplementary Material

Additional file 1 Medicinal plants and fungi used by the people of Mustang district, Nepal. Click here for file
