# Supplementary material for: The use of medicinal plants in the trans-himalayan arid zone of Mustang district, Nepal
Source: J Ethnobiol Ethnomed. 2010 Apr 6;6:14. doi: 10.1186/1746-4269-6-14 (PMC2856531; doi:10.1186/1746-4269-6-14)
Supplement: Additional file 1 — Medicinal plants and fungi used by the people of Mustang district, Nepal. [file 1746-4269-6-14-S1.PDF]

Additional File 1. Medicinal plants and fungi used by the people of Mustang district, Nepal.

Scientific name (Voucher I.D.)	Vernacular name	Part used	Phenology	Ailments treated	C.I.[†]	Methods of preparation	Treatment	Growth form	Other Uses
**Alliaceae**									
**Allium carolinianum* DC. (V4439)	Rotangtea (G)	L	July-Aug.	Child birth, nutrition	+++++	Vegetable	Oral, b.i.d. until cured	H	Spice, pickle
**A. prattii* C.H. Wright apud Forbes & Hemsl. (V3847)	Nyeshing (A)	Bu	June-Aug.	Infection, chronic respiratory diseases	++	Paste, powder with hot water	Oral, s.i.d. until cured	H	Vegetable, spice, pickle
A. wallichii Kunth (V 3878)	Khanakhansha (A)	Wp	Aug.- Sep.	Fever, tonsillitis, cough, cold, loss of appetite	+++++	Water decoction	Oral, b.i.d. until cured	H	Vegetable, spice, pickle
**Amaranthaceae**									
**Amaranthus lividus* L. (V2966)	Bakhrakana (N)	Wp	Apr.-July	Over flow of blood in menstruation & child birth	+	Paste, pills, tablets with hot water	Oral, s.i.d. until cured	H	Vegetable, fodder
**Apiaceae**									
Carum carvi L. (V4468)	Chir (G)	Sd	July-Aug.	Cough, cold, tonsillitis, sinusitis, headache	+++	Water decoction	Oral, s.i.d. until cured	H	Nd
Heracleum candicans Wall. ex DC. (V2908)	Tokar, Neghala (G, N)	Sd	June-July	Cough, cold	++	Water decoction	Oral, t.i.d. until cured	H	Nd
**Araceae**									
Arisaema flavum (Forssk.) Schott (V4477)	Timtry, Tandhun/Dawa (G, A)	Rttu, Fl	May-July	Sinusitis, skin diseases, bone spurs, pregnant woman, blood diseases	+	Powder with hot water	Oral, s.i.d. until cured	H	Vegetable
						Paste	Topical, s.i.d. until cured		
A. jacquemontii Blume (V4492)	Thomo, Dhaba (G, A).	Rttu, Fl	June-Oct.	Sinusitis, skin diseases, bone spurs, pregnant woman, blood diseases	+	Powder with hot water	Oral, s.i.d. until cured	H	Vegetable
						Paste	Topical, s.i.d. until cured		

Scientific name (Voucher I.D.)	Vernacular name	Part used	Phenology	Ailments treated	C.I.[†]	Methods of preparation	Treatment	Growth form	Other Uses
**Asparagaceae**									
Asparagus filicinus Buch.-Ham. ex D. Don (V2075)	Nirshing (G)	Rt	June-Nov.	Pneumonia	+	Powder with milk, honey	Oral, b.i.d. until cured	H	Nd
						Paste	Topical, b.i.d. until cured		
**Asteraceae**									
*Anaphalis nepalensis (Sprengel) Hand.-Mazz. (V4539)	Tacha (A)	Wp	June – Sep.	Fever, edema	++	Powder with hot water	Oral, t.i.d. until cured	H	Incense, decoration
A. triplinervis (Sims) C.B. Clarke (V4522)	Fojormendo, Ta/Tayung (G, A)	Wp	June-Dec.	Edema	++	Rolled ball over a glowing ember	Oral,s.i.d. until cured	H	Nd
Arctium lappa L. (V3894)	Jisung (A)	Fr	Apr.-Oct.	Nerve diseases, kidney stone	+	Powder, pills, tablets with hot water	Oral, s.i.d. until cured	H	Nd
Artemisia biennis Willd. (V3849)	Chaphong (A).	Wp	July-Oct.	Lung fever	++	Water decoction	Oral, s.i.d. until cured	H	Incense, fodder
*A. caruifolia Buch.-Ham. (V4003)	Chaphong, Serphan (A)	L, St	July-Aug.	Cough, cold, lung fever, allergic skin	++	Water decoction	Oral, s.i.d. until cured	H	Incense, fodder
						Paste	Topical, s.i.d. until cured		
*A. dubia Wall. ex Besser (V4302)	Khangkhar (A)	L	July-Oct.	Backbone pain, allergies, skin wounds	++	Paste with hot water	Oral, s.i.d. until cured	H	Incense, fodder
						Paste	Topical, s.i.d. until cured		
*A. gmelinii Weber ex Stechm. (V4133)	Bajha, Fumung (G, A)	Wp	July-Sep.	Nose swelling, ear pain, allergies, skin wounds	+++	Water decoction, paste with hot water	Oral, s.i.d. until cured	H	Incense, fodder
						Paste	Topical & Nasal, s.i.d. until cured		

Scientific name (Voucher I.D.)	Vernacular name	Part used	Phenology	Ailments treated	C.I.[†]	Methods of preparation	Treatment	Growth form	Other Uses
A. indica Willd. (V4134)	Khamba, Khankhar (T/G, A)	Wp	June-July	Sinusitis	+++	Paste with hot water	Oral, s.i.d. until cured	H	Incense, fodder
						Paste	Topical, s.i.d. until cured		
**Aster diplostephioides* (DC.) C.B. Clarke (V4515)	Mara, Motolugmick (G, A)	Fl	July-Sep.	Fever, wounds, chest pain, nerve diseases, anti-poison, stomach & liver diseases	++	Powder with hot water	Oral, b.i.d. until cured	H	Nd
A. stracheyi Hook. f. (V4438)	Mara (G)	Fl	July-Sep.	Fever, wounds, chest pain, anti-poison, nerve, stomach & liver diseases	+	Powder with hot water	Oral, b.i.d. until cured	H	Nd
**Cirsium falconeri* (Hook. f.) Petr. (V4528)	Thakilo, Chyangser (N, A)	Wp, Rt	July-Aug.	Edema, wounds, painful & excessive urination	++++	Water & jaggery decoction	Oral, s.i.d. until cured	H	Nd
						Paste	Topical, s.i.d. until cured		
**Leontopodium monocephalum* Edgew. (V3113)	Kotha (T)	Wp	July-Oct.	Boils	++	Paste	Topical, s.i.d. until cured	H	Nd
Saussurea gossypiphora D. Don (V3867)	Kanglamatho (A)	Wp	July-Oct.	Menstrual disorders & problems, sinusitis, bone fractures, wounds, nose bleeds, stomachache	++	Paste with milk, hot water	Oral, b.i.d. until cured	H	Nd
**S. hookeri* C.B. Clarke (V3229)	Ghanghala (A)	Wp	July-Oct.	Anti-poison, fever, bone fractures	++	Paste with hot water	Oral, b.i.d. until cured	H	Nd
**Senecio diversifolius* Dumort. (V4819)	Mara (G)	Rt	Oct.-Feb.	Fever	++	Water infusion	Oral, t.i.d. until cured	H	Nd
**Sonchus wightianus* DC. (V4282)	Nonechar, Nochare (G, A)	Wp	Apr.-Nov.	Vitamins, bile disorders	+	Powder, paste with hot water	Oral, s.i.d. until cured	H	Vegetable
**Taraxacum eriopodum* (D. Don) DC. (V4012)	Chayathi/ Khurmang (A)	Wp	June-Sep.	Fever	+++	Powder with hot water	Oral, s.i.d. until cured	H	Nd
**T. tibetanum* Hand.-Mazz. (V4421)	Khurmang, Bhaghamugphocho Khurmo, Chayathi/Dhunghip	Rt	June-Sep.	Chronic fever, cough, cold, gastritis, eye diseases, infections, bile diseases, headache,	+++	Water decoction, paste with hot water	Oral, s.i.d. until cured	H	Nd

Scientific name (Voucher I.D.)	Vernacular name	Part used	Phenology	Ailments treated	C.I.[†]	Methods of preparation	Treatment	Growth form	Other Uses
	hol (G, T, A, N)			loss of appetite, diarrhoea, dysentery					
**Berberidaceae**									
Berberis aristata DC. (V4464)	Karya, Kyerwa (G, A)	Fr, Rt, St	Apr.-June	Bile disorders, blood circulation, diarrhoea, dysentery, jaundice, eye diseases	++++	Eaten raw, water decoction	Oral, b.i.d. until cured	S	Edible raw, fence, fuelwood
						Filtered juice	Oral & others, b.i.d. until cured		
**B. ceratophylla* G. Don (V4446)	Kyerpa (G)	Fl, Fr, St	Apr.-Sep.	Edema, nerve dispersed conditions, body pain	++++	Water decoction	Oral, b.i.d. until cured	S	Edible raw, fence, fuelwood
						Paste	Topical, s.i.d. until cured		
**B. koehneana* C.K. Schneid. (V3874)	Kerwa (A)	St	Apr.-Sep.	Eye diseases, bile diseases	+++	Powder with hot water	Oral, b.i.d. until cured	S	Edible raw, fence, fuelwood
**B. mucrifolia* Ahrendt (V4213)	Kerwa (A)	Fl, Fr, St	Apr.- Oct.	Kidney diseases, bile disorders	+++	Water decoction	Oral, 2 × 1 until cured	S	Edible raw, fence, fuelwood
**Betulaceae**									
Alnus nepalensis D. Don (V2977)	Utish (N)	Stbr	May-Oct.	Body pain, chronic fever	++	Powder with hot water	Oral, b.i.d. until cured	T	Fence, fuelwood, fodder, organic manure
**Bignoniaceae**									
Incarvillea arguta (Royle) Royle (V4406)	Tonghshel (A)	L, St, Fl	June-Aug.	Gastritis, high blood pressure	++	Water decoction	Oral, s.i.d. until cured	H	Fodder
**Boraginaceae**									
Arnebia benthamii (Wall. ex G. Don) I.M. Johnst.(V3857)	Dimok, Cithoup (A)	Rt	May-July	Bodypain, lung disease, to increase hair, hemorrhoids	++	Powder with hot water, water decoction	Oral, s.i.d. until cured	H	Fodder, dye, soap

Scientific name (Voucher I.D.)	Vernacular name	Part used	Phenology	Ailments treated	C.I.[†]	Methods of preparation	Treatment	Growth form	Other Uses
						Face wash	Wash, s.i.d. until cured		
**Lindelofia longiflora* (Benth.) Baill. (V3864)	Badhakuro (D)	Rt	June-Aug.	Boils	++	Paste	Topical, b.i.d. until cured	H	Nd
Maharanga emodi (Wall.) A. DC. (V 2298)	Maharangi, Dimock (N, A)	Wp	June-Sep.	High blood pressure	+++	Paste with hot water	Oral, b.i.d. until cured	H	Dye
**Brassicaceae**									
**Arabidopsis himalaica* (Edgew.) O.E. Schulz (V3876)	Chakrakma (A)	Wp	June-Sep.	Leg & backbone pain	++	Juice	Oral, t.i.d. until cured	H	Nd
**Descurainia sophia* (L.) Webb ex Prantl (V4447)	Khangyo, Kapho (A)	Wp	Mar.-May	Cough, cold	+	Powder with hot water	Oral, b.i.d. until cured	H	Nd
**Erysimum hieraciifolium* L. (V 2397)	Pihili Phol (N)	Wp	July-Aug.	Fever	++	Water decoction	Oral, t.i.d. until cured	H	Nd
**Cannabaceae**									
Cannabis sativa L. (V3584)	Kantsya, Bhango (G, T)	Sd, L	June-Sep.	Gastritis, constipation	+++++	Powder smoked with tobacco	Oral, s.i.d. until cured	H	Pickle, psychoactive
**Caprifoliaceae**									
**Lonicera myrtillus* Hook. f. & Thomson (V3892)	Phanghama (A)	L, St, Fl, Sd	June-July	To purify impure blood, overflow of blood during menstrual disorders	++	Paste with hot water	Oral, b.i.d. until cured	S	Edible raw, fuelwood, fence
**L. rupicola* Hook. f. & Thomson (V3875)	Phanghama (A)	L, St, Fl, Sd	June-July	To purify impure blood, overflow of blood during menstrual disorders	++	Paste with hot water	Oral, b.i.d. until cured	S	Edible raw, fuelwood, fence
**Chenopodiaceae**									
**Chenopodium foliosum* (Moench) Asch. (V4459)	Nihaue (A)	Fr	June-Sep.	Edema	+	Water decoction	Oral, s.i.d. until cured	H	Edible raw, vegetable
[§]**Clavicipitaceae**									

Scientific name (Voucher I.D.)	Vernacular name	Part used	Phenology	Ailments treated	C.I.[†]	Methods of preparation	Treatment	Growth form	Other Uses
Cordyceps sinensis (Berk.) Sacc (V3333)	Yartsagumba, Jibanbhuti (A/G, T/N)	Wp	Apr.-Aug.	Periods of fatigue, low energy, tonic	+++++	Milk decoction, powder with honey	Oral, s.i.d. until cured	H	Nd
**Convolvulaceae**									
Convolvulus arvensis L. (V4398)	Shulughawaniwa (A).	Wp	Mar.-Sep.	Cough, cold, typhoid, disease of air, wind	++	Water decoction	Oral, b.i.d. until cured	H	Fodder
**Crassulaceae**									
**Rhodiola quadrifida* (Pall.) Schrenk (V4593)	Chengchungh (A)	Rt	June-Aug.	Asthma, aundice, wounds, anti-poison, lung, kidney & skin disease	+	Powder with hot water	Oral, s.i.d. until cured	H	Nd
**Cupressaceae**									
Cupressus torulosa D. Don (V4301)	Ghuejhokpa, Shukpa (T/G, A)	Fr	Jan.-Nov.	Bodyache, leg pain, sinusitis, gingivitis, mouth swelling	++	Powder, tablets with hot water	Oral, b.i.d. until cured	T	Fuelwood, fence, incense, decoration
						Paste	Topical, s.i.d. until cured		
**Juniperus communis* L. (V3324)	Phar, Chukshar (G, A)	Fr, L	May-Aug.	Kidney diseases	++	Paste with hot water, milk	Oral, t.i.d. until cured	S	Fuelwood, fence, decoration, incense, organic manure
J. indica Bertol. (V3589)	Far, Chhukpha (G, A)	Co	May-Aug.	Tuberculosis	++	Powder with hot water	Oral, s.i.d. until cured	T	Fuelwood, fence, decoration, incense, organic manure
J. squamata Buch.-Ham. ex D. Don (V3872)	Sukri, Chhukshar (G, A)	L, Fr	June-Oct.	Kidney diseases	+	Paste with hot water	Oral, b.i.d. until cured	S	Fuelwood, fence, decoration, incense, organic manure

Scientific name (Voucher I.D.)	Vernacular name	Part used	Phenology	Ailments treated	C.I.[†]	Methods of preparation	Treatment	Growth form	Other Uses
**Elaeagnaceae**									
Hippophae salicifolia D. Don (V2095)	Tarbu, Cheche (G, T)	Fr	May-July	Periods of low energy	+++++	Water decoction	Oral, b.i.d. until cured	T	Eaten raw, fuelwood, fence, pickle
H. tibetana Schltdl. (V 3127)	Tarbu (G)	Fr	Apr.-May	Tonic, cough, cold, fatigue, intestinal worms	++++	Juice	Oral, b.i.d. until cured	S	Eaten raw, fuelwood, fence, pickle
**Ephedraceae**									
Ephedra gerardiana Wall. ex Stapf (V4208)	Somalata, Chaya (G, A)	Rt, Wp	July-Aug.	Chest pain, vertigo/dizziness, headache, respiratory diseases, nose bleeding, cuts, wounds, gastritis	+++++	Powder with hot water, water decoction<br>Paste	Oral, s.i.d. until cured<br>Topical, s.i.d. until cured	H	Fuelwood, fodder
**Ericaceae**									
Rhododendron anthopogon D. Don (V2864)	Palu, Sangalin (G, A)	Fl, St, L	May-July	Loss of appetite, stomach & liver disease, vomiting	+++	Paste, powder with hot water	Oral, b.i.d. until cured	S	Incense
R. arboreum D. Don (V3589)	Gurans (N)	Fl	Feb.-May	Bronchitis, to remove lodged bones or spines out of the tongue	++++	Eaten raw, water decoction	Oral, b.i.d. until cured	T	Incense, fuelwood
**Fabaceae**									
*Argyrolobium roseum (Cambess.) Jaub. & Spach (V2907)	Samraphol, Marbhan, Mushuhang (N, T, A)	Fr	Apr.– Aug.	Fever, communicable diseases	+	Water decoction	Oral, t.i.d. for 2 days	H	Nd
*Caragana brevispina Royle (V4135)	Thangchar(A)	Fl	Apr.-Oct.	Blood diseases, blood purifier, liver diseases, diseases of urine, ribs pain, tonic, allergy	+++	Paste with hot water<br>Paste	Oral, s.i.d. until cured<br>Topical, s.i.d. until cured	S	Edible raw, fodder, fence, fuelwood

Scientific name (Voucher I.D.)	Vernacular name	Part used	Phenology	Ailments treated	C.I.[†]	Methods of preparation	Treatment	Growth form	Other Uses
C. gerardiana Royle (V4405)	Tanglikhtha (A)	Fl, Fr	May-July	Fever	+++++	Powder with hot water	Oral, s.i.d. until cured	S	Edible raw, fodder, fence, fuelwood
C. jubata (Pall.) Poir. (V4041)	Thanglang, bha (T, A)	Fl	June-July	Tonic	++	Eaten raw	Oral, b.i.d. until cured	S	Edible raw, fodder, fence, fuelwood
**Oxytropis williamsii* Vassilcz. (V4388)	Dhanghar, Thakshyakarwa (G/T, A)	Wp	June-Aug.	Allergy, cough, cold, fever	+	Powder with hot water	Oral, b.i.d. until cured	H	Incense
**Gentianaceae**									
Halenia elliptica D. Don (V4235)	Sekwahoha/Tiktha, Jackthick (G/T, A)	Wp	July-Oct.	Pulse rate, bile disorders, fever, fatigue, low energy	++	Water decoction	Oral, b.i.d. until cured	H	Nd
**Swertia macrosperma* (C.B. Clarke) C. B. Clarke (V3898)	Tithe (N)	Wp	June-Sep.	Cough, cold, fever	+	Water decoction	Oral, b.i.d. until cured	H	Nd
**Juglandaceae**									
Juglans regia L. (V5403)	Katutun (G)	Fr	Apr.-May	Cough, cold, chest pain, vitamin	+++	Eaten raw	Oral, b.i.d. until cured	T	Eaten raw, fuelwood
**Lamiaceae**									
**Clinopodium umbrosum* (M. Bieb.) K. Koch (V4358)	Sarsang, Nemayahulo/ Yauckching (G, A)	Wp	Mar.-Oct.	Gastritis, bone fractures	++	Paste, powder with hot water	Oral, s.i.d. until cured	H	Nd
**Isodon rugosus* (Wall. ex Benth.) Codd (V4540)	Mara (G)	Wp	May-Sep.	Gingivitis, tooth pain, sinusitis	+	Powder with milk	Oral, s.i.d. until cured	H	Nd
						Powder	Others, s.i.d. until cured		
Origanum vulgare L. (V2934)	Akhebobo, Ghodhamarcha (G, T)	Wp	June-Aug.	Sinusitis	+++++	Paste with hot water	Oral, s.i.d. until cured	H	Incense

Scientific name (Voucher I.D.)	Vernacular name	Part used	Phenology	Ailments treated	C.I.[†]	Methods of preparation	Treatment	Growth form	Other Uses
						Scent	Others, s.i.d. until cured		
Prunella vulgaris L. (V2869)	Balbhuti (D)	Wp	June-July	Pneumonia	++	Paste with milk, hot water	Oral, s.i.d. until cured	H	Nd
						Paste	Topical, s.i.d. until cured		
**Salvia castanea* Diels (V4592)	Dhurso (N)	Stbr	May-Sep.	Excessive menstrual bleeding, hemorrhage	++	Powder, paste with hot water	Oral, s.i.d. until cured	H	Nd
Thymus linearis Benth. (V3893)	Akheeno, Macto(G, A)	Wp, Rt, L, St, Fl	Apr.-Sep.	Fever, stomachache, anthelmintic, toothache, tooth pain	+++++	Water decoction, paste with hot water	Oral, 1-t.i.d. until cured	H	Nd
**Liliaceae**									
Lilium nepalense D. Don (V2249)	Pana, Rakshimalmal (D, T)	Wp	June-July	Fever	++	Paste with hot water	Oral, s.i.d. until cured	H	Decoration
**Malvaceae**									
Malva verticillata L. (V4188)	Tangshang, Chemba/Chyapa, Khamo (G, A)	Rt, St	Feb.-Aug.	Bone fractures, kidney disease	++++	Powder with hot water	Oral, s.i.d. until cured	H	Organic manure
						Paste	Topical, s.i.d. until cured		
**Menispermaceae**									
**Cissampelos pareira* L. (V3791)	Aalungh, Aalunkha (T, G)	Rt	May-June	Gastritis, constipation	+++++	Juice	Oral, b.i.d. until cured	C	Nd
[§]**Morchellaceae**									
**Morchella esculenta* Pers. (V3310)	Guchichaue (G)	Wp	May-June	Heart diseases	+++++	Confidential to amchi	Oral & others	H	Vegetable
**Nyctaginaceae**									

Scientific name (Voucher I.D.)	Vernacular name	Part used	Phenology	Ailments treated	C.I.[†]	Methods of preparation	Treatment	Growth form	Other Uses
**Mirabilis himalaica* (Edgew.) Heimerl (V4361)	Nigibulung, Khemba/ Bhatu (G, A)	Wp	July-Oct.	Vitamin, edema, cold	++	Paste with hot water	Oral, s.i.d. until cured	H	Nd
**Orchidaceae**									
Dactylorhiza hatagirea (D. Don) Soo (V3839)	Panchaule, Lova (G, A)	Rt	June-July	Snake bite	+++++	Paste	Topical, b.i.d. until cured	H	Nd
**Orobanchaceae**									
Pedicularis longiflora Rudolph (V3897)	Langhanasherpo (A)	Wp	May-Oct.	Headache, bile disorders	+	Paste, tablets with hot water	Oral, b.i.d. until cured	H	Nd
**Papaveraceae**									
**Corydalis govaniana* Wall. (V4318)	Longshil (A)	Wp	May-Aug.	Tonsillitis, fever, bile disorder, allergy	+	Water decoction	Oral, b.i.d. until cured	H	Nd
**Dicranostigma lactucoides* Hook. f. & Thomson (V4417)	Rafendi, Khumang (G, A)	Fl, L	June-July	Bile disorder, headache, diuretic, reduces fat & thins blood in coagulation period	++	Paste, powder tablets, pills, with hot water	Oral, t.i.d. until cured	H	Nd
**Meconopsis staintonii* Grey-Wilson (V3829)	Upalserpo (A)	Rt	June-Sep.	Stomachache, gastritis, diarrhoea, pain near the side of heart	++	Powder with hot water	Oral, s.i.d. until cured	H	Nd
**Phrymaceae**									
Lancea tibetica Hoof. f. & Thomson (V2994)	Payagtsawa (A)	Wp	May-Aug.	Heart & lung diseases, gastritis, anthelmintic	++	Paste with hot water	Oral, s.i.d. until cured	H	Nd
**Pinaceae**									
**Pinus wallichiana* A. B. Jacks. (V3121)	Thansin (G)	Co	Apr.-June	Tuberculosis	++	Powder with hot water	Oral, s.i.d. until cured	T	Fence, fuelwood, decoration, organic manure, construction

Scientific name (Voucher I.D.)	Vernacular name	Part used	Phenology	Ailments treated	C.I.[†]	Methods of preparation	Treatment	Growth form	Other Uses
**Plantaginaceae**									
Neopicrorhiza scrophulariiflora (Pennell) D.Y. Yong (V4445)	Kutki (G)	Rt	June-Aug.	Fever, cough, cold, headache	+++++	Water & Jaggery decoction, powder with hot water, milk, ghee	Oral, b.i.d. until cured	H	Nd
**Plantago erosa* Wall. (V4530)	Naram (A)	Wp	Mar.-Nov.	Diarrhoea, constipation	++	Water decoction	Oral, b.i.d. until cured	H	Nd
**Polygonaceae**									
**Bistorta affinis* (D. Don) Greene (V3129)	Khaldi (G)	Rt, Fl	June-Sep.	Diarrhoea, blood deficiency	++	Water decoction, powder with milk	Oral, b.i.d. until cured	H	Nd
Oxyria digyna (L.) Hill (V3891)	Yupha (A)	Wp	May-June	Constipation, gastritis, diuretic, stomach swelling	+++	Paste with hot water	Oral, s.i.d. until cured	H	Pickle
Rheum australe D. Don (V3267)	Padamchal (N)	Rt	June-Sep.	Malarial fever	+++++	Powder with milk, hot water	Oral, s.i.d. until cured	H	Pickle
Rumex nepalensis Spreng. (V3873)	Hali, Lungso/Somangh (G, A)	Rt, Wp	Apr.-July	Bone fractures, edema, lung diseases, liver disease, joint pain, vomiting, air, wind diseases, to treat dehydration	+++	Paste, powder with hot water, milk	Oral, s.i.d. until cured	H	Vegetable, fence, dye
						Paste	Topical, s.i.d. until cured		
**Primulaceae**									
**Androsace muscoidea* Duby (V4590)	Panghdhum (A)	Wp	May-July	Fever, edema, allergy	+	Paste with hot water	Oral, t.i.d. until cured	H	Nd
						Paste	Topical, s.i.d. until cured		
**A. robusta* (Knuth) Hand.-Mazz. (V3880)	Panghdhum (A)	Wp	May-July	Cough, cold, tonsillitis, allergy	++	Paste, powder with hot water	Oral, s.i.d. until cured	H	Nd
						Paste	Topical, s.i.d. until cured		

Scientific name (Voucher I.D.)	Vernacular name	Part used	Phenology	Ailments treated	C.I.†	Methods of preparation	Treatment	Growth form	Other Uses
**A. tapete* Maxim. (V2511)	Panghdhum (A)	Wp	May-July	Allergic skin	+	Paste, powder with hot water	Oral, s.i.d. until cured	H	Nd
						Paste	Topical, s.i.d. until cured		
**Ranunculaceae**									
Aconitum naviculare (Bruhl) Stapf (V 3861)	Ponkar (T/G/A)	Rt	July-Oct.	Fever, headache, bile & liver diseases	+++++	Paste, pills, tablets with hot water, ghee	Oral, b.i.d. until cured	H	Nd
**A. orochryseum* Stapf (V3862)	Nirmasi, Bonghnama (T/G, A)	Wp, Rt	July-Nov.	Cough, cold, bile disorders, sinusitis, fever, allergy, inappropriate medication	+++++	Paste, powder with milk, hot water	Oral, b.i.d. until cured	H	Nd
						Paste	Topical & nasal, b.i.d. until cured		
**A. spicatum* (Bruhl) Stapf (V3871)	Chandruk, Menchang (T/G, A)	Wp, Rt	Aug.-Oct.	Infected wounds, tonic, boils, fever, allergy, edema, cuts, to counteract the effects of poison	+	Pills, tablets with hot water	Oral, b.i.d. until cured	H	Ritual & religious
						Paste	Topical, b.i.d. until cured		
**Clematis barbellata* Edgew. (V2766)	Laharejhar, Kramay (N, G)	L, St, Fl	May-Oct.	Jaundice	+++	Water decoction	Oral, b.i.d. until cured	C	Fodder
C. tibetana Kuntze (V3114)	Damongnakyo (G)	L	July-Sep.	Constipation, liver diseases	++	Paste with hot water	Oral, s.i.d. until cured	C	Fodder
**C. vernayi* C. Fischer (V4533)	Yaawangma (A)	Wp, L	Apr.-Sep.	Gastritis, warts	+	Water decoction	Oral, s.i.d. until cured	C	Fodder
						Paste	Topical, s.i.d. until cured		
**Ranunculus laetus* Wall. ex D. Don. (V4536)	Kangkhar (A)	Wp	Apr.-June	Sinusitis, bodyache, backbone pain	+	Powder with hot water	Oral, s.i.d. until cured	H	Fodder
						Paste	Topical, s.i.d. until cured		

Scientific name (Voucher I.D.)	Vernacular name	Part used	Phenology	Ailments treated	C.I.†	Methods of preparation	Treatment	Growth form	Other Uses
**Thalictrum alpinum* L. (V3552)	Chakcha/Jackhu (A)	Wp	June-Nov.	Cough, cold, fever, food poisoning, joint swelling, hand leg, joint & backbone pain	++	Paste, powder with hot water	Oral, b.i.d. until cured	H	Nd
						Paste	Topical, b.i.d. until cured		
**Rosaceae**									
**Cotoneaster affinis* Lindl. (V4452)	Phanghama (A)	Rt	Apr.- May	To clear impure blood	+	Paste, powder with hot water, milk	Oral, b.i.d. until cured	S	Edible raw, fence, fuelwood
Fragaria nubicola Lindl. ex Lacaita (V2859)	Safaltang, Shagi (G, A)	Fr	May-Oct.	Diarrhoea	+++	Juice	Oral, b.i.d. until cured	H	Edible raw
**Prunus armeniaca* L. (V4532)	Khurpani, Khambu/Bhala (G/T, A)	Sd	Mar.-Apr.	Vitamins	+	Eaten raw	Oral, t.i.d. until cured	T	Edible raw, sauce
**P. himalaica* Kitam. (V3870)	Khambu (A)	Sd	Mar.-Apr.	Sinusitis,	+	Eaten raw	Oral, t.i.d. until cured	T	Sauce, hair dye, fuelwood
				Hair long & black		Oil	Others, s.i.d. until cured		
Rosa macrophylla Lindl. (V3591)	Seghu (G)	Fl	June-July	Fever, diarrhoea, bile disorders	++	Water decoction	Oral, s.i.d. until cured	S	Edible raw, fence, fuelwood
R. sericea Lindl. (V3597)	Sewa/Saibhamathi, Songhthakpa/Chewa (G, A)	Wp, Fl, Fr	May-Sep.	Cough, cold, fatigue, fever, blood pressure, headache, numbness of limbs, vertigo/dizziness, poor vision, liver diseases, bile disorder, air, wind diseases	+++++	Water decoction, paste, powder with hot water	Oral, b.i.d. until cured	S	Edible raw, fence, fuelwood
**Rubus foliolosus* D. Don (V3567)	Maplan, Kathandakhari, Gatha (G, T, A)	Fr, St	Mar.-May	Pneumonia, fever	+++	Paste with hot water	Oral, s.i.d. until cured	S	Edible raw, fence
**Salicaceae**									
**Salix babylonica* L. (V4001)	Jankchhar, Chyanghama (T, A)	St	Mar.-May	Body pain, excessive menstrual bleeding	++	Paste with hot water, water	Oral, s.i.d. until cured	T	Fodder, fence, fuelwood, ritual

Scientific name (Voucher I.D.)	Vernacular name	Part used	Phenology	Ailments treated	C.I.[†]	Methods of preparation	Treatment	Growth form	Other Uses
						decoction			& religious
**Saxifragaceae**									
Astilbe rivularis Buch.-Ham. ex D. Don (V3763)	Bhadhango, Subkha (D, A)	Rt, Fr	June-Aug.	Digestive	++++	Powder with hot water	Oral, s.i.d. until cured	H	Nd
Bergenia ciliata (Haw.) Sternb. (V2476)	Pakhanved, Kadur (G/N, A)	Rt	Mar.-Apr.	Diarrhoea, digestive, liver diseases, bile disorders, dysentery, red color urine	+++++	Powder with milk, hot water	Oral, b.i.d. until cured	H	Nd
**Scrophulariaceae**									
Verbascum thapsus L. (V3122)	Yugisingh, Dhumaserche (G, A)	Wp, Rt, Fl, Sd	May-Aug.	Wounds, anthelmintic, passing urine, diuretic, over bleeding, infection, edema, burns, stomachache	++	Powder with hot water, water decoction	Oral, t.i.d. until cured	H	Nd
						Paste	Others, s.i.d. until cured		
**Solanaceae**									
Anisodus luridus Link & Otto (V3689)	Langtang, Bajharbang (G, A)	Sd	June-Oct.	Teeth affected by black worms	+++	Powder, scent, smoke	Other, s.i.d. until cured	H	Organic manure
Datura stramonium L. (V4377)	Dhugloma, Thangthungkarpo (D/T, A)	Fr	July-Sep.	Sinusitis	++	Confidential to amchi	Others	H	Pickle, psychoactive
Hyoscyamus niger L. (V2801)	Langtang, Khayalanghla (G, A)	Sd	May-Sep.	Gingivitis, tooth pain	+++++	Powder	Others, s.i.d. until cured	H	Psychoactive
Solanum nigrum L. (V3546)	Kalobhindo (N)	Sd	Most of the year	Pneumonia	+++	Paste with hot water	Oral, s.i.d. until cured	H	Nd
						Paste	Others, s.i.d. until cured		

Scientific name (Voucher I.D.)	Vernacular name	Part used	Phenology	Ailments treated	C.I.[†]	Methods of preparation	Treatment	Growth form	Other Uses
**Tamaricaceae**									
Myricaria rosea W.W. Sm. (V3527)	Angmeo (G)	L, St, Fl	May-Aug.	Lung diseases, asthma	+++	Water decoction	Oral, s.i.d. until cured	S	Fuelwood, incense
**Taxaceae**									
Taxus baccata L. subsp. *wallichiana* (Zucc.) Pilg. (V2489)	Silingi, Jhamarshing (G, T)	Fr	May-Aug.	Cancer	++++	Water decoction	Oral, s.i.d. until cured	T	Fuelwood, furniture, fence, decoration
**Thymelaeaceae**									
Stellera chamaejasme L. (V3124)	Rekemukta, Rechakpa (G, A)	Rt, L, Fl	May-Aug.	Edema, bone fractures	++++	Powder, paste with milk, hot water	Oral, s.i.d. until cured	H	Nd
						Paste	Topical, b.i.d. until cured		
**Urticaceae**									
Urtica dioica L. (V3877)	Sishno, Shaa (N, G)	L	Mar.-July	Chronic diseases, blood pressure, to increase blood, edema, difficulty in passing urine	+++	Vegetable,	Oral, s.i.d. for 1 day	H	Vegetable
						Vapor	Nasal and others, s.i.d. until cured		
**Valerianaceae**									
Nardostachys grandiflora DC. (V4589)	Panghpoi (G)	Wp	June-July	Diarrhoea, fever, gastritis, headache, anthelmintic, edema, rheumatism	+++++	Powder with hot water	Oral, s.i.d. until cured	H	Incense
Valeriana jatamansi Jones (V2398)	Napu, Ghyapo (G, A)	Rt, L	Feb.-May	Headache, cut, wounds, eye diseases, boils, fever	+++++	Water decoction	Oral, b.i.d. until cured	H	Incense
						Paste	Others, s.i.d. until cured		

Scientific name (Voucher I.D.)	Vernacular name	Part used	Phenology	Ailments treated	C.I.†	Methods of preparation	Treatment	Growth form	Other Uses
**Violaceae**									
Viola biflora L. (V2297)	Tamick/Makdawa (A)	Wp	May-July	Boils, wounds, bone fracture, vitamin, loss of appetite, headache	++++	Paste with hot water	Oral, b.i.d. until cured	H	Nd
**Zingiberaceae**									
**Hedychium ellipticum* Buch.-Ham. ex Sm. (V3798)	Kachur (T)	Rt	July-Sep.	Rib pain, respiration	++	Paste with hot water	Oral, t.i.d. until cured	H	Nd
						Paste	Topical, s.i.d. until cured		

Wp: Whole plant, Rt: Root, Fl: Flower, Sd: Seed, L: Leaves, St: Stem, Fr: Fruit, Br: Bark, Stbr: Stem bark, Rttu: Root tuber, Bu: Bulb, Co: Cone, N: Nepali, A: Amchi, G: Gurung, T: Thakali, V: Voucher, H: Herb, S: shrub, T: Tree, C: Climber, Nd: Not documented, s.i.d: once a day, b.i.d.: twice a day, t.i.d.: thrice a day (standard dose is 1/2-1 glass of decoction or infusion, unless otherwise indicated). Pills and tablet forms of medicine are prepared only by amchi.

*Indicates species not previously known for its medicinal use in Mustang.

† Consensus index. Indicates citation by % of informants. +: ≤ 10%; ++: 11-25%; +++: 26-50%; ++++: 51-75%; +++++: ≥ 76%

§ Indicates fungal species.
